# Editorial: Exploring XR technologies for joint surgery: innovations and clinical applications

**DOI:** 10.3389/fsurg.2026.1864293

**Published:** 2026-05-29

**Authors:** Shilong Su

**Affiliations:** Department of Orthopedics, Peking University Third Hospital, Beijing, China

**Keywords:** augmented reality, extended reality, mixed reality, orthopedic, virtual reality

As society ages, the number of patients in the orthopedic field has increased sharply and diseases have become more complex. It is gratifying to note that the development of science and technology has brought assistance to the treatment of diseases. In the past decade or so, extended reality (XR) technology has developed rapidly. From virtual reality (VR), to augmented reality (AR), to mixed reality (MR) with great application value, this has provided a huge boost to the development of medical care. XR interacts between the virtual digital world and the real world, providing an unprecedented immersive experience (1). XR has been widely used in the orthopedic field, especially joint surgery, including intraoperative assistance (2, 3), medical education (4), postoperative rehabilitation (5) and remote consultation. To this end, we have created this research topic to provide a platform for researchers to share their application experience and innovative achievements, and provide reference for the application of XR in the field of joint surgery.

Zhang et al. explored the feasibility of using AR navigation technology to assist atlantoaxial pedicle screw placement. They found that AR navigation technology can well assist the surgeon in completing the surgery and meet the clinical requirements for the efficiency and accuracy of the surgery. The study by Meng et al. focused on the latest MR technology. Unlike VR and AR, MR can realize the interaction of planned virtual guidance images with the real world and realize depth-aware holographic visualization. They summarized the progress of studies on the application of MR in femoral tunnel positioning in anterior cruciate ligament reconstruction, and pointed out that the current clinical evidence is still in its early stages and that there are still multiple limitations that need to be addressed, such as registration drift, occlusion, sterility and workflow integration. Carrasco et al. used a systematic review method to comprehensively evaluate relevant studies on the application of MR in orthopedic surgery over the past decade, and finally included 16 studies. They found that existing clinical evidence mainly demonstrated the technical feasibility and early clinical applicability of MR, but did not provide enough high-quality evidence to support its clinical effectiveness or superiority over traditional surgical methods. Wang et al. combined MR with 3D printing for clinical joint orthopedic teaching. They found that integrating 3D printed models and MR technology into clinical teaching of orthopedic and joint surgery significantly improved students' performance, learning efficiency and overall teaching quality, demonstrating the application potential of MR technology in medical education.

Through the articles in this research topic, we understand that XR technology currently has feasibility and application potential in orthopedic surgical guidance and medical education. But at the same time, we must also admit that the current study and application are still in their early stages and there are still many limitations that need to be resolved. This is also the purpose of this research topic, to provide a platform for researchers to exchange results. Finally, we would like to thank the editorial department, authors and reviewers for their support of this research topic. We hope this research topic can provide readers with more reference and inspiration.
